# Status epilepticus

**DOI:** 10.4103/0972-2327.56312

**Published:** 2009

**Authors:** Ajith Cherian, Sanjeev V. Thomas

**Affiliations:** Department of General Medicine, Medical College, Trivandrum, Kerala, India; 1 Department of Neurology, Sree Chitra Tirunal Institute for Medical Sciences and Technology, Trivandrum, Kerala, India

**Keywords:** Anticonvulsants, barbiturates, lorazepam, midazolam, phenytoin, propofol, refractory status epilepticus, status epilepticus

## Abstract

Status epilepticus (SE) is a medical emergency associated with significant morbidity and mortality. SE is defined as a continuous seizure lasting more than 30 min, or two or more seizures without full recovery of consciousness between any of them. Based on recent understanding of the pathophysiology, it is now considered that any seizure that lasts more than 5 min probably needs to be treated as SE. GABAergic mechanisms play a crucial role in terminating seizures. When the seizure persists, GABA-mediated mechanisms become ineffective and several other putative mechanisms of seizure suppression have been recognized. Early treatment of SE with benzodiazepines, followed if necessary by fosphenytoin administration, is the most widely followed strategy. About a third of patients with SE may have persistent seizures refractory to the first-line medications. They require aggressive management with second-line medications such as barbiturates, propofol, or other agents. In developing countries where facilities for assisted ventilation are not readily available, it may be helpful to use nonsedating antiepileptic drugs (such as sodium valproate, levetiracetam, or topiramate) at this stage. It is important to recognize SE and institute treatment as early as possible in order to avoid a refractory state. It is equally important to attend to the general condition of the patient and to ensure that the patient is hemodynamically stable. This article reviews current knowledge regarding the management of convulsive SE in adults.

## Introduction

Status epilepticus (SE) is a common medical emergency associated with high morbidity, if not mortality. Mortality from SE varies from 3–50% in different studies. In elderly patients, refractory status epilepticus (RSE) may lead to death in over 76% cases.[1] The lifetime prevalence of SE in persons with epilepsy range from 1–16%. Precise epidemiological data for SE are not available for India. It has been estimated that up to 150,000 cases of SE occur annually in the US, with 55,000 associated deaths.[2] The incidence of SE in the US ranges from 6.2–18.3 per 100,000 population. It is essential that all physicians to be able to identify and treat SE promptly and efficiently. Prolonged SE can lead to cardiac dysrhythmia, metabolic derangements, autonomic dysfunction, neurogenic pulmonary edema, hyperthermia, rhabdomyolysis, and pulmonary aspiration. Permanent neurologic damage can occur with prolonged SE.

## Definitions

### Status epilepticus

A landmark meeting in Marseilles in 1962 was the first scientific meeting to be devoted to the topic of SE, and the published proceedings is the first monograph on SE. Gastaut stated that ‘there are as many types of SE as there are types of epileptic seizures’ and defined SE as ‘a term used whenever a seizure persists for a sufficient length of time or is repeated frequently enough to produce a fixed or enduring epileptic condition’.[3] The same definition was retained in the revised classification published in 1970.[4] In the classification of 1981, the definition was modified as: ‘a seizure that persists for a sufficient length of time or is repeated frequently enough that recovery between attacks does not occur.’ This definition emphasizes its prolonged nature and potential to cause serious brain damage but fails to provide operational details such as the precise duration of seizure or number of seizures.

In 1993, the American Epilepsy Society Working Group on Status Epilepticus had put forward an operational definition of SE as ‘seizure lasting more than 30 min or occurrence of two or more seizures without recovery of consciousness in between.’[5] The rationale for stipulating this duration was the observations in experimental models that any seizure that persists for more than 30 min is accompanied by serious metabolic decompensation and permanent neuronal damage. Accordingly, most of the protocols for treatment of SE were designed to prevent prolongation of seizures beyond 30 min.

In the past 10 years, there has been considerable rethinking about the precise duration that a seizure must last for it to be designated as SE. The duration of what is accepted as SE has been shrinking progressively. From 30 min specified in the guidelines of the Epilepsy Foundation of America's Working Group on Status Epilepticus it was reduced to to 20 min; the Veterans Affairs Status Epilepticus Cooperation Study stipulated 10 min and, most recently, a length of 5 min has been proposed. Most seizures cease within a minute or two and if the seizure is prolonged beyond a few minutes, it is unlikely to stop by itself. Video-EEG analysis of 120 secondarily generalized tonic–clonic seizure (GTCS) in 47 patients has shown that the maximum duration was 108 s (range 16–108 s, mean 62 s). Primary generalized seizures were of shorter duration than secondarily generalized seizures.[6] Spontaneous termination becomes less likely in seizures lasting >5 min, and the longer the seizure continues, the more difficult it is to control the seizure with antiepileptic drugs (AEDs) and the greater the degree of neuronal damage. It appears that in SE the innate inhibitory mechanisms in the brain that put a halt to the seizure are no longer effective. A duration of 5 min probably is a reasonable cutoff to distinguish isolated seizures from SE. Numerous clinical studies have demonstrated a relation between seizure duration and mortality.[7] When confronted with a patient with continuous seizures, one cannot wait for 30 min, or for that matter even for 15 min, before initiating therapy. Further, there is evidence that seizures may become refractory and difficult to control if treatment is delayed. Lowenstein et al.[8] have proposed that SE be defined as a continuous, generalized, convulsive seizure lasting >5 min, or two or more seizures during which the patient does not return to baseline consciousness. Probably any convulsive seizure that lasts more than 2 min deserves to be managed as SE.

### Refractory status epilepticus

About 9–31% of patients with SE may fail to respond to standard treatment. This subgroup of RSE has greater morbidity and mortality. RSE is defined as continuous or repetitive seizures lasting longer than 60 min despite treatment with a benzodiazepine (lorazepam) and another standard anticonvulsant (usually phenytoin/fosphenytoin) in adequate loading dose.[9]

Malignant SE is a severe variant of RSE, in which the seizure fails to respond to aggressive treatment with even anesthetic agents. It typically occurs in young patients (18–50 years) in the setting of encephalitis.

## Classification of status epilepticus

Semiologically and electrophysiologically there are several types of seizures; these have been broadly classified as either generalized or partial seizures by the International League Against Epilepsy. In principle there can be as many types of SE as there are types of seizures. This has led to complex classifications of SE. However, using electroclinical features, SE may be classified broadly as convulsive SE and nonconvulsive SE.

### Convulsive status epilepticus

Convulsive SE (CSE) can be further classified into (a) tonic–clonic SE, (b) tonic SE, (c) clonic SE and (d) myoclonic SE. Generalized tonic–clonic SE is the most common form of SE. Myoclonic SE presents as a bilateral massive myoclonus, along with polyspike discharges on EEG, and usually carries a good prognosis. But the myoclonic status that follows severe hypoxic-ischemic insult, viral encephalitis, and prion disease is associated with poor prognosis. This review deals predominantly with generalized tonic–clonic SE, which is the most commonly observed form of SE in clinical practice.

### Nonconvulsive status epilepticus

Nonconvulsive SE (NCSE) refers to continuous or near-continuous generalized electrical seizure activity lasting for at least 30 min, but without physical convulsions. CSE may evolve into the nonconvulsive form after treatment or NCSE may arise de novo. NCSE is characterized by abnormal mental status, unresponsiveness, ocular motor abnormalities, persistent electrographic seizures, and possible response to anticonvulsants.[9] All patients with prolonged postictal confusion or unexplained coma should undergo EEG monitoring for confirmation.[10] NCSE has long been divided into two main categories: absence SE (ASE) and complex partial SE (CPSE). Until recently most cases of NCSE were considered to be ASE, and CPSE was thought to be a rarity. Several investigators have shown that CPSE is more common than previously thought and that many cases of ASE are actually cases of CPSE that have generalized. Tomson et al. reported on 32 consecutive NCSE patients, most of who had CPSE, and suggested that in adults most cases of NCSE are in fact partial in onset. The distinction between ASE and CPSE is an important one, as ASE is usually easier to treat and may not be associated with significant neuronal damage.

## Epidemiology

It has been estimated that up to 150,000 cases of SE occur annually in the US, with 55,000 associated deaths.[2] Geography, sex, age, and race influence the epidemiology of SE. An incidence of 6.2–18.3 per 100,000 population has been reported in the US.[12] Regardless of geographic influences, SE appears to be more frequent among men, blacks, and the aged.[13] The incidence of SE in the elderly population is at least twice that in the general population.[14] SE in the elderly is of great concern because concurrent medical conditions often exist that are likely to complicate therapy and worsen the prognosis.[15]

### Etiology

Nearly a quarter of persons presenting with SE have preexisting epilepsy. A dramatic drop in serum levels of AEDs due to noncompliance or other reasons is the most common mechanism of SE in such instances. In many patients with a preexisting seizure disorder, no obvious precipitating factor can be identified for the occurrence of SE. SE is more common in patients with secondary generalized epilepsy than in those with idiopathic generalized epilepsy. The causes of SE may differ according to the age at presentation [Table 1] and geographic location. For example, central nervous system (CNS) infections may predominate in children and cerebral malaria can be a common cause in malaria endemic area.[16] In patients with SE and CNS infection, 24.3% had a refractory status that was associated with a high mortality.[17]

**Table 1:** Causes of status epilepticus according to age[18]

Causes	Children	Adults
Infection	35.7	6
Medication changes	20	18
Unknown	9	8
Metabolic precipitants	8	9
Congenital precipitants	7	-
Anoxia	5	12
CNS infection	5	6
Trauma	3.5	4.5
Alcohol/drugs	-	13
Stroke	-	25

More than half of SE episodes occur in patients without a history of prior seizures. Genetic factors may also play a role as twin studies have demonstrated a greater concordance in monozygotic as opposed to dizygotic twins. Cerebral toxoplasmosis, lymphoma, and anticonvulsant withdrawal are important causes of SE in HIV infection. Alcohol intoxication is a major precipitant in alcohol-dependent patients. A variety of drugs and medications may reduce the seizure threshold or increase the clearance of AEDs and predispose to SE [Table 2].

**Table 2:** Commonly used drugs that may predispose to status epilepticus by lowering the seizure threshold or by increasing the clearance of antiepileptic drugs

Antibiotics (especially in older adults or in patients with renal impairment)
Penicillins
Imipenem
Cephalosporins
Isoniazid
Metronidazole
Erythromycin
Ciprofloxacin, ofloxacin
Antihistamines
Diphenhydramine
Antipsychotics
Clozapine, chlorpromazine
Antidepressants
Maprotiline
Bupropion
Tricyclics, especially clomipramine
Other drugs
Fentanyl
Flumazenil
Ketamine
Lidocaine
Lithium
Meperidine
Propoxyphene
Theophylline
Baclofen (acute withdrawal)

## Pathophysiology

Clinical and experimental studies have shown that SE evolves through an initiation phase to a maintenance phase. In the initiation phase the triggering stimuli evoke discrete seizures that tend to abate as soon as the stimulus is removed. In the subsequent maintenance phase discrete seizures coalesce together into a continuum, with triggering stimuli no longer required to sustain a train of seizures. The intensity and duration of the stimulation has a direct influence on the transition from the initiation phase to the maintenance phase. A variety of signaling molecules such as GABA-A (γ-aminobutyric acid) antagonists, glutamate agonists, cholinergic (muscarinic) agonists, tachykinins, galanin antagonists, and opiate k antagonists have been found to be involved in the initiation phase. In contrast to this, much less is known about the maintenance phase. In fact only a limited number of molecules have been found that block the maintenance phase (e.g., NMDA (N-methyl-D aspartate) antagonists, substance P antagonist, galanin, and dynorphin).

The evolution of a single seizure to SE depends on several factors. In experimental electrogenic SE, at least 30 min of stimulation is required to produce self-sustained SE. The limbic system is clearly at increased risk for injury during SE and, due to its nature and connections with the rest of the brain, it may play a crucial role in generating seizures. It has been hypothesized that within the limbic system, the dentate gyrus acts as a ‘gatekeeper’ and prevents excitatory stimulation from spreading through the hippocampus until a point of maximal dentate activation is reached. Once this point is exceeded, excitatory inputs can spread through the hippocampus and may then propagate to involve widespread neocortical areas.

It is likely that ineffective recruitment of inhibitory neurons, together with excessive neuronal excitation, plays a role in the initiation and propagation of the electrical disturbance occurring in SE. GABA is the major inhibitory neurotransmitter in the CNS. It is released from GABA minergic neurons and binds to several types of GABA receptors [i.e., GABA-A (GABA type A), GABA-B, and GABA-C receptors]. GABA receptors are macromolecular proteins that form a chloride ion channel complex and contain specific binding sites for GABA and a number of allosteric regulators, including barbiturates, benzodiazepines, and a number of anesthetic agents. GABA receptor–mediated inhibition may be responsible for the normal termination of a seizure. In addition, the activation of the NMDA receptor by the excitatory neurotransmitter glutamate may be required for the propagation of seizure activity. The activation of NMDA receptors results in increased levels of intracellular calcium, which may responsible for the nerve cell injury seen in patients with SE.[19,20] A growing body of evidence from research and clinical observation supports the concept that SE becomes more difficult to control as its duration increases. It has been postulated that this may occur due to a mechanistic shift from inadequate GABAergic inhibitory receptor–mediated transmission to excessive NMDA excitatory receptor–mediated transmission.[21–23] This phenomenon has important clinical implications. Benzodiazepine-like drugs, which potentiate GABAergic inhibition, have an important role in the early initiation phase of SE but they may prove ineffective in the advanced stages of SE, when NMDA antagonists have the potential to be beneficial. Prolonged epileptiform bursting results in a reduction of GABA-mediated synaptic inhibition. Furthermore, it is seen that constitutive internalization of GABA-A receptors is rapid and accelerated by the increased neuronal activity associated with seizures, and inhibition of neuronal activity reduces the rate of internalization. These findings suggest that the rate of GABA-A receptor internalization is regulated by neuronal activity and its acceleration contributes to the reduction of inhibitory transmission observed during prolonged seizures. The GABA-A agonist diazepam is less effective in ameliorating SE when administered after perforant path stimulation than when administered before. When diazepam was given 60 min before perforant path stimulation, seizures were virtually eliminated.[22]

In humans and experimental animals, sustained seizures cause selective neuronal loss in vulnerable regions such as the hippocampus, cortex, and thalamus. The degree of neuronal injury is closely related to the duration of seizures, underscoring the importance of rapid control of SE. Meldrum and Brierley[24] and Nevander *et al*.[25] have demonstrated that even without attendant hypoxia, acidosis, hyperthermia, or hypoglycemia, ongoing seizures in primates and rats can cause neuronal death. Wasterlain *et al*.[26] reported neuronal loss in the hippocampus and other brain regions in patients with nonconvulsive SE who did not have preexisting seizures or systemic abnormalities. Neuron-specific enolase, a marker for acute neurologic injury, has been demonstrated to be increased in patients with nonconvulsive SE who did not have preceding or coexistent cerebral injury.[27] Neuronal death is probably caused by the release of excitatory neurotransmitters.

In animal models, the most effective agents in aborting the maintenance stage of SE are NMDA blockers, substance P antagonists, galanin, and dynorphin. Maladaptive changes such as increased substance P and decreased galanin may contribute to the maintenance of SE. Possible mechanisms contributing to termination of SE may include neuronal injury, depletion of metabolic stores, depletion of excitatory neurotransmission, or late enhancement of inhibitory mechanisms.

## Stages of Status Epilepticus

SE may be broadly divided into two stages [Table 3].[28] The first stage is characterized by generalized convulsive tonic–clonic seizures that are associated with an increase in autonomic activity, resulting in hypertension, hyperglycemia, sweating, salivation, and hyperpyrexia. During this phase, cerebral blood flow is increased due to increased cerebral metabolic demands. After approximately 30 min of seizure activity, patients enter the second phase, which is characterized by the failure of cerebral autoregulation, decrease in cerebral blood flow, increase in intracranial pressure, and systemic hypotension. During this phase, electromechanical dissociation may occur in which, although electrical cerebral seizure activity continues, the clinical manifestations may be restricted to minor twitching.

**Table 3:** Systemic and cerebral pathophysiological changes associated with convulsive status epilepticus

Stage of compensation (< 30 min)	Stage of decompensation (> 30 min)
Increased cerebral blood flow	Failure of cerebral autoregulation
Cerebral energy requirements matched by supply of O2 and glucose	Hypoglycaemia
Increased glucose concentration in the brain	Hypoxia
Increased catecholamine release	Acidosis
Increased cardiac output	Hyponatremia
	Hypo/hyperkalemia
	Disseminated intravascular coagulation
	Leukocytosis
	Falling blood pressure
	Falling cardiac output

SE may pass through five distinct electrographic stages: (a) discrete electrographic seizures, (b) merging of electrographic seizures - waxing and waning of ictal discharges, (c) continuous ictal discharges, (d) continuous ictal discharges punctuated by flat periods, and (e) periodic epileptiform discharges against a relatively flat background.[27]

## Diagnosis

Occasionally, the diagnosis of SE can be difficult. Fulminant tonic–clonic movements with frothing, and dysautonomic features with loss of consciousness are important pointers to the diagnosis of SE. However, there are several other conditions that may mimic SE at some point of time [Table 4].

**Table 4:** Mimics of generalized convulsive status epilepticus

Generalized convulsive SE
Pschogenic status epilepticus
Decerebrate spasm
Tetanus
Malignant hyperthermia
Neuroleptic malignant syndrome
Paroxysmal dyskinesia
Acute ballismus or chorea
Status dystonicus
Tremor
Tetany
Clonus
Shivering
Periodic limb movement of sleep
Myoclonus
Partial SE
Hemifacial spasm
Asymmetric tremor
Myoclonic jerks
Palatal myoclonus
Tic disorder
Focal dystonia
Paroxysmal nocturnal dyskinesia
Blepharospasm
Nonconvulsive SE
Akinetic rigidity
Locked-in syndrome
Akinetic mutism
Catatonia
Atonic disorders
Periodic paralysis
Cataplexy
Cognitive disorders
Encephalopathy
Encephalitis
Amnesia
Sleep disorders
Psychiatric disorders

Psychogenic SE must be differentiated from true SE.[11] The diagnosis must be reached quickly, as delay in recognition can lead to iatrogenic complications due to aggressive treatment. The diagnosis may be difficult in patients with subtle writhing and in-phase limb movements and unresponsive behavior. Video-EEG monitoring is the gold standard to differentiate the mimickers.

Diagnosis of NCSE can be challenging. It may not be considered in patients who present in altered sensorium or coma following a convulsion. All comatose patients should therefore be carefully examined for evidence of minor twitching, which may involve the face, hands, or feet or may present as nystagmoid jerking of the eyes. Continuous EEG monitoring can be helpful in such instances. Towne and colleagues[29] evaluated 236 patients with coma and no overt seizure activity and found that 8% of the patients had NCSE, as determined by EEG monitoring. Therefore, it is essential that an urgent EEG be performed in patients with unexplained coma.

## Treatment

SE is one situation where emergent symptomatic relief (control of seizures) takes precedence over systematic evaluation. The principles of treatment are protection of the vital functions, particularly cardiorespiratory functions, and early and energetic treatment of convulsions with appropriate anticonvulsants. This needs to be followed by systematic evaluation of the cause of SE and steps to prevent complications and recurrence of seizures [Algorithm 1].

**Algorithm 1:**
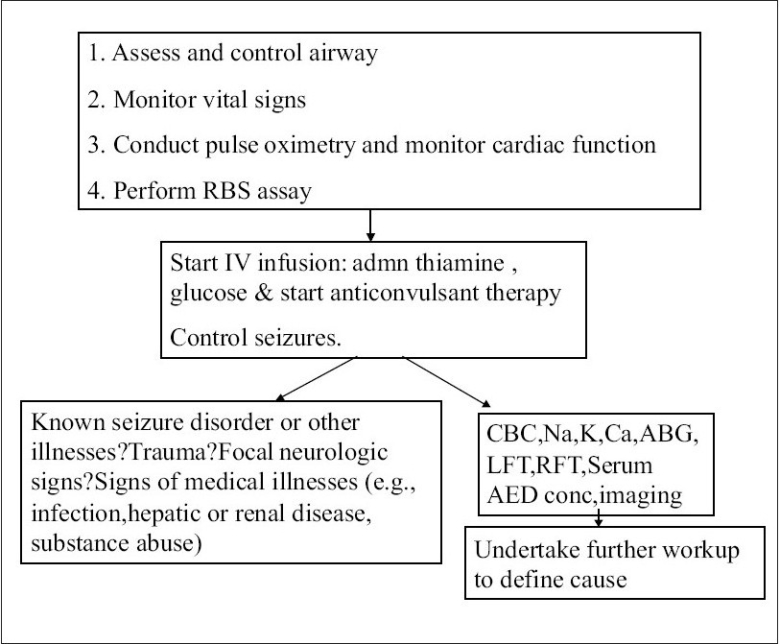
The initial management of SE

### General measures

As with any critically ill patient, the first step in the management of a patient with SE would be to ensure an adequate airway and to provide respiratory support. The patient should be positioned such that self-injury is prevented during seizure activity. Earlier teaching was that patients should be rolled onto their side during a seizure. Recent indications demonstrate that this may cause more harm than good. Patients who have been rolled onto their side during a major motor seizure are at greater risk for self-injury, such as a dislocated shoulder. As patients are not breathing during a generalized tonic–clinic seizure, they are not at high risk for aspiration until the event ends. Immediately following the seizure, patients usually take a deep breath. Therefore, the patient should be rolled over onto his or her side immediately after the motor activity ceases. Likewise, to prevent injury to the patient, suction of the oropharynx can wait until the end of the seizure.

To avoid injuries to oral structures, one should resist the urge to forcibly introduce a tongue depressor or other hard objects between locked teeth. Gentle suction can be applied to remove saliva and blood if any. Two large-gauge intravenous (IV) catheters should be inserted to facilitate fluid resuscitation and pharmacotherapy. Should peripheral venous access be difficult, the placement of a central venous catheter is recommended. Despite the periods of apnea and cyanosis that occur during the tonic or clonic phases of the seizure, most patients in SE breathe sufficiently well as long as the airway remains clear. An oral airway may be required once the seizure has terminated to prevent airway obstruction. Once the seizures are controlled, and if the patient is oxygenating and ventilating adequately, endotracheal intubation may not be required for airway protection, even if the patient remains comatose.[30] However, in this situation precautions should be taken to avoid aspiration, and a nasogastric tube should be placed to ensure that the stomach is empty. Endotracheal intubation will be required in patients who continue to experience seizures despite receiving first-line therapy. There is no available data as to the pharmacologic agents that are preferred for achieving endotracheal intubation. Most of the patients who require endotracheal intubation will be comatose or would have received lorazepam or some other benzodiazepine. Hence a hypnotic agent may not be required for intubation. In some situations, an anesthetic induction dose of propofol or midazolam may terminate the seizure activity and facilitate intubation. Neuromuscular blockade will be required to facilitate intubation if the patient continues to have tonic–clonic seizure activity despite these pharmacologic interventions. Rocuronium (1 mg/kg), a short-acting, nondepolarizing muscle relaxant that has little effect on blood pressure and intracranial pressure is the preferred agent.[31] Succinylcholine should be avoided, if possible, as some of these patients with prolonged convulsions may be prone to hyperkalemia secondary to rhabdomyolysis. Prolonged neuromuscular blockade should be avoided as far as possible.

Hypoglycemia must be excluded rapidly in all patients presenting with SE and corrective measures must be instituted if serum glucose is low (<60 mg%). If prompt measurement of blood glucose level is not possible, the patient should receive 100 mg of thiamine intravenously followed by a 50 ml bolus of 50% dextrose. Blood pressure (BP), electrocardiography (ECG), and core temperature should be monitored. If the patient develops significant hyperthermia (i.e., temperature >40°C), passive cooling is required.[32] Blood specimens should be obtained for laboratory examinations [Table 5]. Continuous motor seizures may lead to muscle breakdown, with the release of myoglobin into the circulation. The maintenance of adequate hydration is necessary to prevent myoglobin-induced renal failure. Forced saline solution diuresis and urinary alkalinization should be considered in the presence of myoglobinuria or significantly elevated serum creatine kinase levels (i.e., > 5,000 to 10,000 U/l).

Once the seizures are controlled, imaging of the brain (CT scan and/or MRI) and cerebrospinal fluid (CSF) examination will be useful in patients who do not have a past history of a seizure disorder. It must be emphasized that the first priority is to control the seizure. Endotracheal intubation and induction of neuromuscular paralysis for the sole purpose of facilitating imaging of the patient may increase morbidity and is strongly discouraged.

**Table 5:** Blood investigations in a patient with status epilepticus

Random blood sugar
Electrolytes - sodium, potassium, calcium, magnesium
Complete blood count
Renal function test, liver function test
Antiepileptic drug level
Arterial blood gas

The American Academy of Neurology and the Practice Committee of the Child Neurology Society has provided some guidelines for the assessment of a child with SE. AED levels should be measured when a child with treated epilepsy develops SE (level B). There is insufficient data to support or refute recommendations as to whether blood cultures or lumbar puncture should be done on a routine basis in children in whom there is no clinical suspicion of a systemic or CNS infection (level U). Toxicology studies and metabolic studies for inborn errors of metabolism may be considered in children with SE when clinically indicated or when the initial evaluation reveals no obvious etiology (level C). An EEG may be considered in a child with SE as it may be helpful in determining whether there are focal or generalized epileptiform abnormalities; this information may guide further investigations into the etiology of SE, especially when there is suspicion of pseudo SE (nonepileptic SE) or NCSE, and may also guide treatment (level C). Neuroimaging may be considered after the child with SE has been stabilized if there are clinical indications or if the etiology is unknown (level C). There is insufficient evidence to support or refute routine neuroimaging in a child presenting with SE (level U).[33]

### Pharmacotherapy

Since only a small fraction of seizures go on to become SE, the probability that a given seizure will proceed to SE is small at the start of the seizure but increases as the seizure duration increases. If a seizure lasts > 5 min, clinical experience suggests that the likelihood of spontaneous termination decreases. The goal of pharmacologic therapy is to achieve rapid and safe termination of the seizure and to prevent its recurrence, without adverse effects on the cardiovascular and respiratory systems or alteration of the level of consciousness. Benzodiazepines (diazepam, lorazepam, midazolam) and hydantoin (phenytoin, fosphenytoin) are the first-line drugs for termination of SE. These drugs have different pharmacodynamic and pharmacokinetic properties, which determine the rapidity of their clinical effect, their efficacy in terminating SE, and their duration of action [Table 6]. Benzodiazepines carry the disadvantage of excessive sedation, which may interfere with subsequent monitoring of the patient. The benzodiazepines bind to the benzodiazepine binding site on the GABA receptor complex, increasing GABAergic transmission, while the barbiturates act directly on the GABA receptor. The antiseizure activity of phenytoin is complex; however, its major action appears to the blocking of voltage-sensitive, use-dependent sodium channels.

**Table 6:** Pharmacokinetic parameters of commonly used drugs for status epilepticus[34]

	Latency (in minutes)	Duration (in hours)
Lorazepam (IV)	3–10	12–24
Diazepam (rectal)	5–15	<1
Diazepam (IV)	1–5	<1
Midazolam (IM)	5–10	<1
Midazolam (buccal)	5–10	<1
Midazolam (IV)	10–30	12–2
Phenytoin (IV)	10–30	12–24
Fosphenytoin (IV)	10–30	12–24
Phenobarbitone (IV)	5–30	48–72
V (IV)	<20	8–24

Latency = Time interval between commencement of infusion and onset of action. Duration = The time, from onset of infusion, for which the drug may maintain adequate anticonvulsant level in the blood.

### Prehospital treatment of SE

The longer an episode of SE continues, the more refractory to treatment it becomes and the greater is the likelihood of complications. More prolonged seizures carry higher risk of mortality;[8] hence it would be useful to initiate treatment for SE even as the patient is being transported to the hospital. Paramedics and ambulance staff, if trained appropriately, could administer preliminary treatment even before shifting the patient to the hospital and save valuable time.

Two randomized trials have been conducted in prehospital settings, comparing buccal midazolam with rectal diazepam for the treatment of continuing seizures in children and adolescents. The midazolam dose is usually 0.2–0.3 mg/kg IM/intranasal/buccal (in children) or 10 mg in an adult. The onset of action is within 1–5 min. Buccal midazolam was found to be equally effective or better than rectal diazepam in terminating seizures, without causing any increase in adverse cardiorespiratory events or need for ventilation. Drug delivery options include intranasal, intramuscular, and rectal routes. Thus, early intervention, without the need for intravenous access and ventilator support, could prevent the emergence of RSE. Administration of midazolam by the buccal or intranasal routes is easier and more socially acceptable than the rectal route and allows patients to treat themselves, especially in cases with prolonged auras, simple partial seizures, or clusters with recovery between seizures. It is superior to intravenous or rectal benzodiazepines, primarily because of the ease with which it can be administered. Buccal or nasal preparations of midazolam are now readily available.

### In-hospital treatment of SE

The drug of choice for in-hospital treatment of SE is intravenous lorazepam. Publication of the Veterans Affairs (VA) Cooperative Trial in 1998[35] and the San Francisco Emergency Medical Services Study in 2001[36] allows for an evidence-based approach to the choice of the first-line agent for use in terminating SE.

The VA Cooperative Study randomized 384 patients with overt generalized SE into four intravenous treatment arms: (a) lorazepam, 0.1 mg/kg; (b) diazepam, 0.15 mg/kg, followed by 18 mg/kg phenytoin; (c) phenytoin, 18 mg/kg; and (d) phenobarbital, 15 mg/kg. Successful treatment required both clinical and EEG termination of seizures within 20 min of the start of therapy and no seizure recurrence within 60 min from the start of therapy. Patients who did not respond to the first treatment received a second choice of treatment drug and, if necessary, a third choice. The latter choices were not randomized. SE was terminated in 64.9% of patients randomized to lorazepam, 58.2% of those randomized to phenobarbital, 55.8% of those randomized to diazepam and phenytoin, and 43.6% of those randomized to phenytoin (*P* = 0.002 for lorazepam *vs* phenytoin). There was no difference between the treatment arms in recurrence rates.

The San Francisco Emergency Medical Services Study[36] was a randomized, double-blind trial to evaluate IV benzodiazepine administration by paramedics for the treatment of out-of-hospital patients with SE. In this study, 205 patients were randomized to receive IV diazepam (5 mg), lorazepam (2 mg), or placebo. An identical second injection was administered if needed. SE had terminated at arrival in the emergency department in 59.1% of the patients treated with lorazepam, in 42.6% of the patients treated with diazepam, and in 21.1% of patients treated with placebo. The duration of SE was shorter in the lorazepam group compared to the diazepam group.

Current evidence indicates that lorazepam administered intravenously in out-of-hospital settings by paramedics or in emergency room settings is superior to diazepam and phenytoin in abolishing SE in a short period of time. The pharmacokinetic properties of lorazepam favor its use over that of diazepam. The anticonvulsant effect of a single dose of diazepam is very brief (20 min), whereas that of lorazepam is much longer (>6 h); also, the risk of respiratory depression may be greater with diazepam. Although diazepam has a much longer elimination half-life, it is rapidly redistributed from the brain to the peripheral fat stores due to its high lipid solubility, which accounts for its shorter anticonvulsant activity. Clonazepam has also been used in the initial drug management of GCSE and has been compared in clinical trials with diazepam and lorazepam. After intravenous loading clonazepam had clinical efficacy similar to other two drugs. When compared with lorazepam, improvement in the EEG was quicker with lorazepam and the clinical symptoms resolved more completely with clonazepam.[37]

Lorazepam should be stored in light-proof containers and should be restocked every 4–6 months.[36] Although refrigeration is recommended for lorazepam (but not for diazepam), Gottwald and coworkers[38] have demonstrated that lorazepam retains 90% of its original potency even when stored without refrigeration in ambulances for as long as 5 months. Several groups recommend that phenytoin or fosphenytoin (20 mg/kg) be given following the administration of lorazepam. While there is no data to demonstrate that the administration of phenytoin following the use of lorazepam increases the response rate, it may be reasonable to use it in situations where recurrent seizures requiring AED therapy are anticipated (e.g., acute encephalitis).

IV fosphenytoin (a prodrug of phenytoin without the propylene glycol carrier) is the best second-line therapy in SE. It is quickly dephosphorylated to phenytoin when given IM or IV. Fosphenytoin has several advantages over phenytoin. It can be infused using standard intravenous solutions, whereas phenytoin should not be given in dextrose-containing fluids (because of drug precipitation). It can be given intramuscularly. Fosphenytoin can be infused at a faster rate (150 mg/min) than phenytoin, which carries some risk of cardiac arrhythmia and hypotension, especially in the elderly. Intramuscular administration of fosphenytoin is well tolerated and cardiac monitoring is not required. It has fewer adverse effects than phenytoin. Pruritus in the inguinal region during fosphenytoin infusion is not an allergic reaction and does not require stoppage of the drug (although slowing the rate may help decrease the itching). This itching is most likely due to the phosphate load. Serum phenytoin levels should be obtained only > 2 h after an IV load or > 4 h after IM to allow complete conversion to phenytoin.

According to the VA study, only half of the enrolled patients responded to the first line of treatment, with cessation of seizures within 20 min of drug administration. This indicates that about half of the patients who present in SE will require additional treatment.

### Continuous EEG monitoring in refractory status epilepticus

Continuous EEG (CEEG) monitoring offers several advantages in the management of SE. It is useful in patients who do not recover consciousness once the convulsive seizure has been aborted. In a study of SE patients who had remained in altered sensorium after CSE,[39] 48% had continued electrographic seizures and 14% had persistent NCSE.

Abnormal eye movements (nystagmoid eye jerks, hippus, repeated blinking, and persistent eye deviation) in a patient who has had neurosurgical intervention, brain tumor, or past history of stroke or meningitis is a good pointer for NCSE. Abnormal eye movements under such circumstances have high sensitivity (100%) but low specificity (55%). An emergent EEG evaluation would be useful in such circumstances.

The goal regarding the activity pattern on the EEG remains a matter of debate. There is no prospectively collected evidence that a burst-suppression EEG pattern is required for, or indicative of the termination of SE. Many patients may achieve complete seizure control when the EEG background reveals continuous slow activity. EEG must be checked hourly once the patient achieves a stable response to the selected drug. When CEEG is used, the primary endpoint for therapy is suppression of electroencephalographic spikes. It may be necessary to continue therapy or increase dosage until EEG shows a burst-suppression pattern with short interburst intervals of < 1 s (secondary endpoint), provided the patient is hemodynamically stable. There is less chance for breakthrough seizures when the EEG shows burst suppression rather than slow activity.

It is useful to regularly check blood levels of phenytoin or other AEDs to ensure that adequate levels are maintained throughout the therapy. In a resource-poor nation, frequent blood level monitoring may not be always feasible. Interaction between different drugs, as well as systemic disorders and organ dysfunctions, can lead to unexpected fluctuations in blood levels of AEDs.

## Management of Refractory Status Epilepticus

About 30% of SE may prove resistant to standard treatment with one of the benzodiazepines and phenytoin. In the VA cooperative study,[35] 55% of patients with generalized CSE did not respond to first-line therapy. Probably RSE is rather underrecognized. This subgroup of patients has higher risk of complications and extended hospital stay and mortality. Most of them have some underlying structural cerebral damage or metabolic disorders or cerebral hypoxia. Patients with NCSE and focal motor status are more likely to enter the phase of RSE. Other risk factors include delay in receiving treatment, metabolic encephalopathy, hypoxia, and encephalitis.

It is important to recognize that very few patients who failed to respond to a first-line drug are likely to respond to an alternate drug from among the first-line drugs. In the VA study,[35] the aggregate response rate to a second drug from the first-line agents was 7% and to a third drug it was only a meager 2.3%. Furthermore, the data from that study suggest that the administration of further doses of lorazepam will not be useful. If seizures continue after administration of lorazepam and fosphenytoin in appropriate dosages, a provisional diagnosis of RSE needs to be made. Such patients would require admission to an intensive care unit for close monitoring and more aggressive treatment under assisted ventilation.

Facilities for endotracheal intubation and ventilation may not be available in most of the peripheral hospitals in India and other developing countries. An alternate regimen that would not require administration of muscle relaxants, endotracheal intubation, and ventilation would be handy in such situations. Recently, new regimens that avoid assisted ventilation have been tried in the management of SE. Their mechanisms of action are different from that of benzodiazepines and phenytoin, which depend upon GABAergic inhibition and sodium channel blocking, respectively.

### Intravenous valproate

Sodium valproate is a broad spectrum AED that has been in clinical use for several decades. But only recently has it been evaluated as an intravenous preparation for the treatment of SE. Intravenous valproate (IV VPA) is a therapeutic option for those patients with cardiorespiratory impairment, ‘do not ventilate’ status, and myoclonic SE. It is a nonsedating drug with an attractive safety profile and is easy to use. IV VPA is well tolerated by even unstable patients with SE. In a recent trial,[40] 68 patients with CSE were randomized to receive either IV VPA (30 mg/kg) or phenytoin (18 mg/kg); Those who received IV VPA had significantly higher rate of cessation of seizures (66%) when compared to the IV phenytoin group (42%). In each group, those who failed to respond were given the other drug as second-line treatment. As the second agent, VPA was effective in 79%, while phenytoin was effective in only 25%. Tolerability did not differ between the two groups. There was no hemodynamic instability. IV VPA had an overall efficacy of 63.3% and rapid administration was well tolerated. It was efficacious even when used as the second, third, or fourth drug.

Intravenously administered valproate is found to be effective against different types of SE, including partial onset, nonconvulsive, absence, and myoclonic SE. The standard loading dose for intravenous administration is 25 mg/kg; nevertheless, in emergent situations, higher doses of 30–60 mg/kg can be used. It is well tolerated at relatively fast infusion rates of 5–6 mg/kg/min. Experience with IV VPA in the treatment of SE is too limited to allow recommendation of its use as a first-line agent. Contraindications for its use include severe liver dysfunction, thrombocytopenia, and active bleeding. The target serum level of valproate in SE ranges from 70 to 140 μg/ml. Its main advantages are the lack of sedation and the fact that intubation for assisted ventilation can be avoided.

### Intravenous levetiracetam

Levetiracetam is another relatively new AED that has a potentially important role in the management of RSE. It is available as an intravenous preparation that can be infused rapidly without the necessity of ventilator assistance. However, its efficacy has not yet been demonstrated through randomized control trials. In some of the pioneering studies, seizure control has been achieved in patients with RSE by administration of levetiracetam (3000 mg/day). It is currently approved for intravenous use in a dosage of up to 3000 mg given over 15 min as replacement for oral dosing (1:1 ratio) in patients who are on levetiracetam and had developed SE. Higher doses of 2500 mg given in 5 min and 4000 mg over 15 min have been found to be well tolerated and safe in healthy volunteers.[41]

### Oral topiramate

Topiramate is another new AED with a mechanism of action that is different from that of the benzodiazepines and other first-line AEDs. When administered by nasogastric tube in doses ranging from 300–1, 600 mg/day, it was effective in aborting RSE. In a small study conducted in children with RSE, seizures were terminated with topiramate given in a loading dose of 10 mg/kg/day followed by a maintenance dose of 5 mg/kg/day.[42]

Newer drugs such as levetiracetam and topiramate have a potentially important place in the management of SE. Their mechanism of action is independent of GABA-mediated inhibition, which tends to become ineffective beyond the first few minutes of SE. These AEDs are well tolerated even at rapid infusion rates and may not aggravate multiple organ dysfunction. They can be administered without ventilator assistance as they cause little sedation. Nevertheless, their safety and efficacy in controlling SE need to be confirmed through well-designed controlled trials.

### Management with ventilator assistance

Patients with RSE who have not responded to the first-line treatment will require admission to the intensive care unit for more aggressive management under assisted ventilation. A reappraisal of the metabolic status and the potential underlying disorder that provoked the RSE is often necessary at this point. Continuous EEG monitoring can be helpful at this stage. Arterial and central venous access is necessary. Continuous infusion of phenobarbital, midazolam, propofol, or pentobarbital, preferably under continuous EEG monitoring, can be tried. The electroencephalographic objective of therapy is the burst-suppression pattern, which needs to be continued for 12 h after the last seizure. Infusion of the anesthetic agent can be reduced every 3 h with EEG monitoring and if there are no clinical or EEG seizures, the patient can be weaned off the ventilator. If seizure recurs, the same agent to which it had responded earlier must be restarted, with weaning attempted after 12 h.[43] No difference in mortality was found between the groups treated with different regimens. Mortality was related to the patient's age and duration of SE rather than the choice of AED. There was lower a frequency of acute treatment failure and breakthrough seizures in those receiving pentobarbital. It should, however, be pointed out that this recommendation is based on limited clinical data, with just over 100 cases of treatment with these agents having been reported.

### Phenobarbital

Phenobarbitone is a long-acting barbiturate that acts by potentiation of GABA and by interfering with sodium and potassium transport across the cell membrane. It is found to be effective in patients who have failed lorazepam or fosphenytoin, with or without valproate. The loading dose is 20 mg/kg IV, with a maximum infusion rate of 50–100 mg/min. The elimination half-life is 72 h. Contraindications include severe liver dysfunction. The main side effects are respiratory depression (patients will often require intubation and ventilation), prolonged sedation, allergy (including Stevens-Johnson syndrome), and blood dyscrasias. The target serum level is 30–45 μg/ml initially.

### Propofol

Propofol is an alkylphenol (2,6-diisopropylphenol) that has been used extensively for the induction and maintenance of anesthesia and for sedation in the intensive care unit. Propofol is a global CNS depressant. It directly activates the GABA receptor.[44] In addition, propofol inhibits the NMDA receptor, modulates calcium influx through slow calcium ion channels, and has antioxidant activity. The excellent pharmacokinetics and favorable adverse effect profile makes propofol the ideal drug to treat RSE. The two main advantages of propofol are a rapid onset and short duration of action. Propofol is a highly lipophilic agent with a large volume of distribution. This property results in its rapid uptake and elimination from the CNS, thereby giving it rapid onset of action and allowing rapid recovery upon discontinuation. Recovery is rapid even after prolonged use. Propofol is metabolized by glucuronidation and sulfate conjugation, and does not accumulate even after long-term infusion. No dose reduction is required in patients with hepatic or renal disease Concomitant IV benzodiazepines allows use of a lower and safer dose of propofol, i.e., the benzodiazepine infusion is used as a propofol ‘dose-sparing’ technique. Benzodiazepines, when administered concomitantly with IV propofol, have a dose-sparing effect on propofol and so lower doses can be equally effective.

The usual loading dose ranges from 3–5 mg/kg but loading doses as high as 10 mg/kg have been safely used. This should be followed by a maintenance infusion at the rate of 30–100 μg/kg/min, which should be titrated to burst-suppression pattern. SE stops within 10 min in most situations. After 12 h of seizure suppression, the dose can be gradually reduced by 50% over the next 12 h and if there is no relapse it can be withdrawn slowly over the subsequent 12 h. If seizures relapse during the weaning period, a further loading dose of 1–3 mg/kg can be administered and the maintenance infusion can be continued until a 12-h seizure-free period occurs.[45]

The most severe complication associated with propofol is the ‘propofol infusion syndrome.’[46] It is a rare complication that occurs when the dosage exceeds 5 mg/kg/h for more than 48 h. The propofol infusion syndrome is a potentially fatal condition characterized by severe metabolic acidosis, hyperlipidemia, rhabdomyolysis, and cardiovascular collapse.[47] It appears that this disorder is due to interference with mitochondrial respiration. The full-blown syndrome tends to occur only in those individuals with a genetic susceptibility. However, the risk appears to be higher in children.[48] It is currently recommended that the dosage of IV propofol should not exceed 100 μg/kg/min in adults. Other contraindications include allergy to soybean oil, egg lecithin, or glycerol. It should be used with caution in combination with carbonic anhydrase inhibitors such as zonisamide and topiramate due to the risk of refractory acidosis. Hyperlipidemia and creatine kinase levels can be useful early markers of this syndrome.

### Midazolam

Midazolam is a fast-acting, water-soluble benzodiazepine with a half-life of 4–6 h. It acts by binding to GABA-A receptors. Midazolam is an alternative to propofol. The drug undergoes hepatic transformation into an active metabolite that is cleared by the kidneys. Midazolam is typically started after securing endotracheal intubation and ventilator assistance. It is usually started with a loading dose of 0.2 mg/kg, but increments of 0.2–0.4 mg/kg can be given every 5 min until the seizures stop or a maximum of 2.9 mg/kg is reached. The maintenance dose is 0.1–0.2 mg/kg/h, given as an infusion to maintain electrographic suppression of seizures. For breakthrough seizures, an additional bolus dose can be given and the continuous IV infusion rate can be increased by approximately 20%. The antiepileptic effect of midazolam lasts from minutes to hours. The reported failure rate with midazolam is 14–18%.

One of the major disadvantages of midazolam is tachyphylaxis, because of which the dose often has to be increased several fold to maintain seizure control. Furthermore, with prolonged infusion, midazolam accumulates in the body, which may result in a prolonged time to awakening.[49]

High-dose barbiturate therapy is a treatment option in RSE. This regimen is often associated with hemodynamic instability and immune paresis. Due to these side effects, high-dose barbiturate therapy is reserved for those patients who do not respond to midazolam or propofol infusion. Intravenous pentobarbitone and thiopentone are two regimens that are often tried. Pentobarbitone has lower lipid solubility than thiopentone. Hence it takes a longer time to achieve its peak effect and it shows better correlation between serum concentrations and clinical effect, especially after prolonged infusion. Therefore most institutions prefer pentobarbitone for induction and maintenance of drug-induced coma in patients who have failed propofol/midazolam. The loading dose is 5 mg/kg; boluses of 5 mg/kg can be repeated until seizures stop. The maximum bolus rate is 25–50 mg/min (depending on the blood pressure). The usual maintenance dose is 0.5–10 mg/kg/h, traditionally titrated to suppression-burst on EEG.

### High-dose phenobarbital

Very high doses of phenobarbital at accumulated daily doses up to 80 mg/kg, with a resulting serum level of more than 1000 μmol/l, has been shown to be effective in achieving seizure control in children with RSE. High-dose phenobarbitone therapy can be considered in patients with RSE who get breakthrough seizures when midazolam or propofol is being tapered off after achieving short-term electrocerebral suppression.

Phenobarbital has no anticonvulsant ceiling effect. Patients with seizures who were treated with phenobarbital at serum concentrations of > 300 mg/l have benefited from further dose increases. The sedative and respiratory-depressant properties of phenobarbital are subject to tolerance over a relatively short time period, but the anticonvulsant activity is not. Therefore, serum concentrations as high as 200–250 mg/l have been maintained in patients, with preservation of respiratory drive and adequate minute ventilation. The aryl moiety of the drug confers potent anticonvulsant properties at doses below values that depress the brainstem reticular formation and consciousness. This relatively high therapeutic index allows nonanesthetic doses of the drug to be effective. The high phenobarbital doses administered to treat SE can thus be gradually withdrawn until consciousness returns. Further dosage reductions to maintain serum drug levels between 20 and 40 mg/l may be considered for chronic, outpatient therapy of generalized epilepsy. Initiation of high-dose phenobarbital therapy is the key to safe withdrawal of pentobarbital.

### Role of inhalational anesthetics

Inhalational anesthetics offer an alternative approach to the treatment of RSE. The advantages include efficacy, rapid onset of action, and the ability to titrate the doses according to the effects demonstrated on the EEG. Isoflurane and desflurane are the two agents that have been tried in the treatment of RSE because of the safety associated with their long-term administration. Isoflurane is the agent of choice for RSE. Isoflurane undergoes significantly less metabolism in the liver than halothane. Both inhalational agents can cause dose-dependent reduction in blood pressure due to peripheral vasodilation and an inotrope and/or vasopressor may be required during their administration, besides adequate fluid resuscitation

Regardless of seizure type, isoflurane and desflurane have consistently stopped epileptic discharges with adequate, sustained electrographic burst suppression within minutes of initiation of therapy. Prolonged use of inhalational anesthetics is well tolerated.

### Neurosurgical treatment of refractory status epilepticus

In selected cases of RSE that have failed to respond to medical management, surgical intervention can be considered as a last resort. This is particularly applicable to cases with an underlying surgically remediable lesion.

## Maintenance Therapy

It is important to ensure that adequate maintenance therapy is instituted simultaneously with the emergent treatment to prevent relapse of SE. Patients already receiving AEDs will require some dose escalation or adjustment according to the prevailing blood drug levels. In patients who are naïve to AEDs, one of the AEDs that had successfully terminated the seizures will have to be continued. If additional medication is needed, the most appropriate AEDs are topiramate and levetiracetam as these drugs can be started at high doses with a low risk of idiosyncratic reactions. A patient who develops SE in the course of ethanol withdrawal may not need AED therapy once the withdrawal has run its course. On the other hand, patients with a new, ongoing, epileptogenic insult (e.g., encephalitis) may require high dosages of AEDs to control their seizures.

## Special Situations

Certain medical conditions can present with SE. The management of SE in such situations may need special approaches.

Porphyria can occasionally be complicated with SE. Most of the enzyme-inducing AEDs such as phenobarbitone, phenytoin, carbamazepine, and lamotrigine are contraindicated in porphyria as they increase the porphyrin production. SE in these cases can be treated with parenteral magnesium sulphate or IV lorazepam without aggravating porphyria. Levetiracetam is a new AED that can be tried in this situation. High carbohydrate and hematin intake tends to reduce porphyrin production and thereby ameliorate the situation. When SE is due to intoxication with isoniazid, administration of high doses of pyridoxine can be useful. Seizures associated with overdoses of tricyclic antidepressants and antimuscarinic agents may respond to physostigmine. Pyridoxine will be especially useful for suspected pyridoxine-dependent seizures.

Aggressive treatment with anticonvulsants and plasma exchange are useful for thrombotic thrombocytopenic purpura complicated by SE. For eclamptic seizures, parenteral magnesium sulphate could be used initially. Benzodiazepine can also be given without causing a depressant effect on the fetus. Phenytoin loading may be pursued with a lower dose (10 mg/kg IV) since there is reduced protein binding in pregnancy; subsequently, 5 mg/kg can be given as a second dose.

## Treatment of Underlying and Precipitating Causes

Comprehensive management of SE includes treatment of precipitants and causes such as noncompliance with AED therapy, acute infections, high fever, hypoglycemia, electrolyte imbalance, organ dysfunction, drug intoxication, poisoning, alcohol withdrawal, excess use of alcohol, stroke, trauma, and hypertensive encephalopathy. These causes must be actively pursued and treated.

## Complications

Patients with SE are prone to several medical complications. Prolonged seizures can lead to multiple organ dysfunctions. Medications such as pentobarbitone may depress the immune response of the subjects and thereby predispose them to infections. Non-neurological complications include nosocomial and ventilator-associated pneumonia, atelectasis, adult respiratory distress syndrome, neurogenic pulmonary edema, pulmonary embolism, hypovolemia, myocardial dysfunction, hypertension, arrhythmias, stress ulcer, gastrointestinal bleed, constipation, diarrhea, paralytic ileus, renal dysfunction, urinary tract infection, and vascular catheter–related sepsis. It is important to watch for these complications so as to detect them as early as possible and institute prompt treatment.

## Pitfalls in Management

SE often fails to respond or gets protracted because of deficiencies in the management. Common errors include misdiagnosis, wrong route of administration (intramuscular phenytoin), inadequate dosing of AEDs, delay in switching to a second-line drug, delay or failure in initiating maintenance therapy with appropriate drugs, failure to detect and treat the precipitating or underlying causes and complications of SE, and delay in providing cardiorespiratory support with endotracheal intubation and vasopressor administration.

## Prognosis

SE has a variable prognosis. In large hospital-based studies, mortality varies from 3–50%. The factors associated with high mortality include refractory seizures, acute symptomatic etiologies (e.g., hypoxia or central nervous system infections), impairment of consciousness, longer duration of SE, and older age (> 70 years). Cardiovascular decompensation during SE, medical complications, and overtreatment with AEDs may also predispose to excess mortality. Coma with recurrent electrographic status and multiorgan dysfunction carries poor clinical outcome. Seizure duration was the single major predictor of mortality on multivariate analysis in one study, with a duration < 10 h resulting in lower mortality (10%) and a duration > 20 h in high mortality (85%). Outcome in RSE is poor; mortality is almost 50%, and only a minority of patients (primarily those with preexisting epilepsy and no acute brain process) return to their premorbid functional baseline.

## Conclusions

SE is a medical emergency that need to be evaluated and managed in a systematic manner. Patients who have persistent generalized seizures beyond 5 min deserve to be treatedt as SE. It is important to initiate treatment as soon as the patient is observed, preferably in the prehospital phase itself in order to prevent resistance to medications. Those patients who have failed to respond to two of the first-line drugs (lorazepam and fosphenytoin in most instances) need to be managed as RSE. Such patients should be moved to intensive care services, where they could be administered second-line drugs (barbiturates or benzodiazepines), with ventilator support and continuous EEG monitoring. If there is no facility for intubation, they can be treated with nonsedating medications such as intravenous valproate, levetiracetum, or oral topiramate. It is equally important to attend to the general medical condition of the patient, even as the AEDs are being administered.
